# Australian Patient Preferences for Discussing Spiritual Issues in the Hospital Setting: An Exploratory Mixed Methods Study

**DOI:** 10.1007/s10943-023-01767-x

**Published:** 2023-02-20

**Authors:** Megan C. Best, Kate Jones, Frankie Merritt, Michael Casey, Sandra Lynch, John A. Eisman, Jeffrey Cohen, Darryl Mackie, Kirsty Beilharz, Matthew Kearney

**Affiliations:** 1 Institute for Ethics and Society, University of Notre Dame Australia, Broadway, PO Box 944, Sydney, NSW 2007 Australia; 2 The School of Medicine Sydney, University of Notre Dame Australia, Sydney, Australia; 3 Garvan Institute of Medical Research, Sydney, Australia; 4 St Vincent’s Private Hospital, Sydney, Australia; 5 Excelsia College, Sydney, Australia; 6 St Vincent’s Hospital, Sydney, Australia; 7 Faculty of Medicine, University of NSW, Kensington, NSW Australia

**Keywords:** (3–5) spirituality, Spiritual history, Patient preferences, Patient–clinician interaction, Healthcare professionals

## Abstract

While there is high patient acceptance for clinical staff discussing issues regarding spirituality with hospital inpatients, it is not clear which staff member patients prefer for these discussions. This unique exploratory study investigated inpatient preferences regarding which staff member should raise the topic of spirituality. A cross-sectional survey was conducted with inpatients at six hospitals in Sydney, Australia (*n* = 897), with a subset invited to participate in qualitative interviews (*n* = 41). Pastoral care staff (32.9%) were the preferred staff members with whom to discuss spiritual issues, followed by doctors (22.4%). Qualitative findings indicated that individual characteristics of the staff member are more important than their role.

## Introduction

In a recent literature review of highly cited definitions of spirituality in the healthcare field, spirituality has been defined as (1) an individual, dynamic human characteristic; (2) which is expressed through beliefs, practices, and experiences in the search for connection with something that promotes meaning and personal growth; and (3) leads to the development of values and positive inner feelings (de Brito Sena et al., 2021). One popular example defines spirituality as ‘a dynamic and intrinsic aspect of humanity through which persons seek ultimate meaning, purpose, and transcendence, and experience relationship to self, family, others, community, society, nature, and the significant or sacred. Spirituality is expressed through beliefs, values, traditions, and practices’ (Puchalski et al., 2014).

Spirituality should be distinguished from religion, in that spirituality is defined as something broader, which may involve religiosity, but goes beyond it. Commonly identified sources of connection are those with family, a higher being or the transcendent, with nature or culture, or with the person’s inner sense of self (Sulmasy, 2002). Health crises trigger existential questions which often require spiritual answers; therefore, it is important that the spiritual needs of patients are addressed in the hospital context and their connection to sources of spiritual strength maintained (Best et al., 2014a).

Spiritual Care Australia is the professional association for spiritual carers (also known as chaplains or pastoral care professionals) in Australia. They describe spiritual care as ‘encompassing all the ways in which attention is paid to the spiritual dimensions of life. It offers a way for people to experience and make meaning of their hopes and fears’ (Spiritual Care Australia, 2021). Interest in spiritual care in healthcare is growing, and awareness increasing of the significant effect of spirituality on physical, mental and social health (Koenig, 2015) and of its potential to strengthen healthcare relationships (Best et al., 2014a, 2014b, 2014c, 2014d) through holistic patient care.

There is a high level of acknowledgement by healthcare providers (HCP) that spiritual care is part of their role, for example, by doctors (Best et al., 2016a, 2016b), nurses (McSherry & Jamieson, 2011; Ross & McSherry, 2018), social workers (Hughes et al., 2018), and physiotherapists (Turner & Cook, 2016). However, this acknowledgement is not reflected in the amount of care provided (Best et al., 2016a, 2016b).

Previous studies have indicated that there are high levels of patient willingness to discuss spirituality, by which we mean issues such as sources of spiritual strength, spiritual wellbeing and spiritual needs (Best, et al., 2015b; Lobb et al., 2019; Snowden et al., 2018). A recent Australian survey commissioned by the Spiritual Health Association reported that 82% of those who had previously received spiritual or pastoral care in hospital believed it helped them to feel comforted by their religious or spiritual beliefs and 54% participants overall would be interested in receiving spiritual or pastoral care in the future hospitalisations; interestingly, higher proportions were found in younger age groups (McCrindle, 2021). However, it is not clear which staff member is preferred by hospital patients for such conversations.

We aimed to investigate the most acceptable person-centred way to assess spirituality and spiritual care needs in Australian hospitals, as a preliminary stage of developing a culturally sensitive history-taking tool for Australian healthcare. A previous paper from this study reported Australian patient preferences as to how they would like to be asked about their spirituality. It found that there was high patient acceptance for discussing spiritual issues in healthcare, but that relevance depended on context (Best et al., 2022a, 2022b).

This paper describes the aspect of the study which aimed to identify the preferred clinician to ask these questions and why.

## Methods

### Research Design

This was an exploratory mixed-method cross-sectional study comprised of a short survey and semi-structured qualitative interviews.

### Participants

Participants were recruited from six hospitals across Sydney, Australia. Hospitals included public and private facilities, which provided both acute and sub-acute inpatient and outpatient care, and represented over 1,000 beds. All but one of the hospitals was administered by a Roman Catholic Organisation. Eligible patients were adult; alert, oriented to person, place and time; able to give verbal consent; able to understand and speak English; and able to complete the survey either personally or with assistance from the researcher. Participants were identified by the Registered Nurses in charge of each ward of the study hospitals. They conducted a clinical assessment to determine which patients met the inclusion criteria. Potential participants were approached by a researcher who asked whether they were willing to participate in a short survey, explaining what it involved and answering any questions. Verbal consent was obtained before the survey was distributed and documented by the return of the anonymous survey (implied consent). Participants were surveyed between March and September 2019.

In accordance with Best Practice Guidelines (QOL Office, 2019), researchers were trained by the senior investigator to administer the questionnaires in a standardised manner to maximise completion rates and reduce the potential for response bias. This involved following a checklist of instructions covering the processes of informed consent, non-coercive survey administration, and recording of results.

The survey contained an invitation to participate in a qualitative interview to explain individual survey responses. If the patient expressed interest in participating in an interview, information about the process was given and the opportunity to ask questions provided. After written consent was received, patient contact details were recorded so that an interview could be arranged, if required, at a convenient time. From those who volunteered, participants were purposively selected to ensure a demographically heterogeneous sample. Ethics approval was granted by the Human Research Ethics Committee at St Vincent’s Hospital, Sydney (HREA AU/1/B78D25).

### Procedure

All participants were asked to complete a survey which included the following questions:

*Demographic details:* age, gender, education level and main lifetime occupation (proxy for socioeconomic level), indigenous status, religion, and length of stay (proxy for severity of illness).

*Preferred member of staff:* Patients were asked to nominate which member of the hospital healthcare team they would prefer to have ask them about spiritual issues that affected them during hospitalisation.

Participants were also asked questions about their preferred language for discussing spiritual issues. The full questionnaire for this study can be found in online Appendix 1.

A subset of patients was invited to complete a qualitative interview. Those who agreed to participate were either interviewed on the ward or scheduled for a telephone interview within 1–2 weeks of giving consent.

Interviews were conducted by two researchers (KB and KJ) and continued until data saturation (no new information after three consecutive interviews). Interviewers asked patients to give examples of spiritual discussions during their hospital admission, identify which staff members were involved, and explain the rationale for the answers they gave in the survey. Participants were therefore asked about spiritual issues that affected them during hospitalisation in the context of the responses they had previously given regarding their understanding of spirituality and their preferred terminology.

### Analysis

*Quantitative:* Demographic data were tabulated, and descriptive statistics generated to describe the results. A series of Fisher’s exact tests were conducted to investigate differences between categorical variables on patient staff member preferences. Associations with sex, age, patient diagnosis, and religious affiliation were examined. Effect sizes were measured using Cramer’s V and considered to be ‘small’ if < 0.3.

*Qualitative:* Interviews were recorded and transcribed verbatim by a professional transcription company. Free text responses to the questionnaires and interview transcripts underwent thematic analysis according to the six stages outlines by Braun and Clarke (Braun & Clarke, 2006): familiarisation with the data; generating initial codes; searching for themes; reviewing themes; defining and naming themes; and producing the report. Familiarisation with the data was achieved by the reading and re-reading of the transcripts by MB, FM and KJ. Initial codes were generated by all three researchers independently using line-by-line coding. The authors then met to compare and review these codes. A code book was created with code definitions and illustrative quotations from the transcripts. These preliminary codes were then used to synthesise groups of data into focused codes, which were applied to further transcripts. Key themes were identified by the first author (MB) and then reviewed by all authors, defined and named. Analysis continued until thematic saturation was reached (no new data in three consecutive interviews). Rigour was derived from six successive rounds of discussion and development of themes until coding was complete. Differences were resolved through discussion and negotiated consensus, therefore allowing rigour and reflexivity in the analysis. As the interviews involved discussion of the participant’s survey results, triangulation of data was achieved through comparison of qualitative and quantitative results (data source triangulation).

The different disciplinary backgrounds (palliative care, social work, psychology and theology) brought to these discussions by the research team allowed for reflection on the role of our individual perspectives in the interpretation of the data.

## Results

### Quantitative

The survey was completed by 897 patients. Response rate was 95%. Thirty patients (3.2 per cent) required assistance in completing the survey. Fifty-two per cent were male. The age range was 20 to 99 years, and the median age was 66.0 years. When asked whether they considered themselves ‘spiritual’, 475 (52.9%) patients agreed or strongly agreed. When asked whether they considered themselves ‘religious’, 345 (38.4%) agreed or strongly agreed.

The largest religious group was Protestant (36%), followed by Roman Catholic/Orthodox (28%) with no religion reported for 26%. This reflects a slightly more religious sample than the last Australian census figures report (39% no religion in 2021) and also a disproportionately larger number of protestants, which could be due to the local population. One-third considered themselves to be neither spiritual nor religious. Patients were admitted to a wide range of hospital wards. Full demographic details are found in Table 1.

**Table 1:** Participant demographic characteristics.n = 897

Demographic items	Category	N (%)
Gender	Female	421 (46.9)
	Male	469 (52.3)
	Missing	6 (0.7)
Age (n,%)	20–29	60 (6.7)
	30–39	57 (6.4)
	40–49	62 (6.9)
	50–59	115 (12.8)
	60–69	173 (19.3)
	70–79	224 (25.0)
	80 and over	126 (14.0)
	Missing	80 (8.9)
Type of patient group (n,%)	Medical	338 (37.9)
	Rehab	84 (9.4)
	Palliative care	55 (6.1)
	Surgical	232 (25.9)
	Emergency Medicine	64 (7.1)
	Geriatric/Aged Care	35 (3.9)
	Psychiatry	10 (1.1)
	ICU	21 (2.3)
	Other	21 (2.3)
	Maternity	32 (3.6)
	Missing	5 (0.6)
Religious affiliation (n,%)	Protestant	326 (36.3)
	Roman Catholic/Orthodox	254 (28.3)
	None	237 (26.4)
	Jewish	35 (3.9)
	Other religions*	39 (4.4)
	Missing	6 (0.7)
I am a spiritual person	Strongly disagree	31 (3.5)
	Disagree	271 (30.2)
	Neither agree nor disagree	99 (11.0)
	Agree	283 (31.5)
	Strongly agree	192 (21.4)
	Missing	21 (2.3)
I am a religious person	Strongly disagree	98 (10.9)
	Disagree	374 (41.7)
	Neither agree nor disagree	57 (6.4)
	Agree	167 (18.6)
	Strongly agree	178 (19.8)
	Missing	23 (2.6)
I am a religious or spiritual person	Not spiritual or religious	269 (30.0)
	Spiritual but not religious	147 (16.4)
	Spiritual and religious	322 (35.9)
	Neither agree nor disagree	40 (4.5)
	Religious but not spiritual	21 (2.3)
	Missing	98 (10.9)
First staff preference to talk about spirituality	Doctors	201 (22.4)
	Nurses	16 (1.8)
	Spiritual carers	295 (32.9)
	Counsellor/Psych	19 (2.1)
	Social Worker	29 (3.2)
	Aboriginal Liaison	1 (0.1)
	Other**	295 (32.9)
	Missing	41 (4.6)

*Other religions included Buddhism, Islam, Hindu, and Indigenous spirituality. ** *Other [preference to talk about spirituality]* included: preference based on personality rather than role of staff member; preference to discuss spirituality with non-staff members, including family, friends, community religious leaders and others with a similar healthcare experience; preference to limit spiritual issues to self-exploration; and those who did not identify an individual but merely did not think discussion of spirituality relevant to a healthcare setting

When asked for first staff preference for discussing spiritual issues that affected them during hospitalisation, most frequently selected were spiritual care staff (32.9%) and then doctors (22%). Two hundred and ninety-five (32.9%) patients selected ‘Other’. There were 137 free text comments from this group which indicated: that the staff member with whom they would like to discuss spiritual issues that affected them during hospitalisation was based on personality rather than role; non-hospital staff would be the person of choice (e.g. family, friends, community spiritual leaders); patient would like to speak to someone with a similar disease experience; patients preferred to be self-reliant; or discussion of spiritual issues was considered irrelevant to the healthcare setting. Non-selection of a staff member did not correlate with not wanting to be asked about spiritual issues. Fisher’s exact test identified no significant associations with participant sex. Some significant associations were found between age and religious affiliation, and staff member preferences but effect sizes were small (< 0.3) and therefore excluded from the analysis (see Table 1).

### Qualitative

**Fig. 1:**
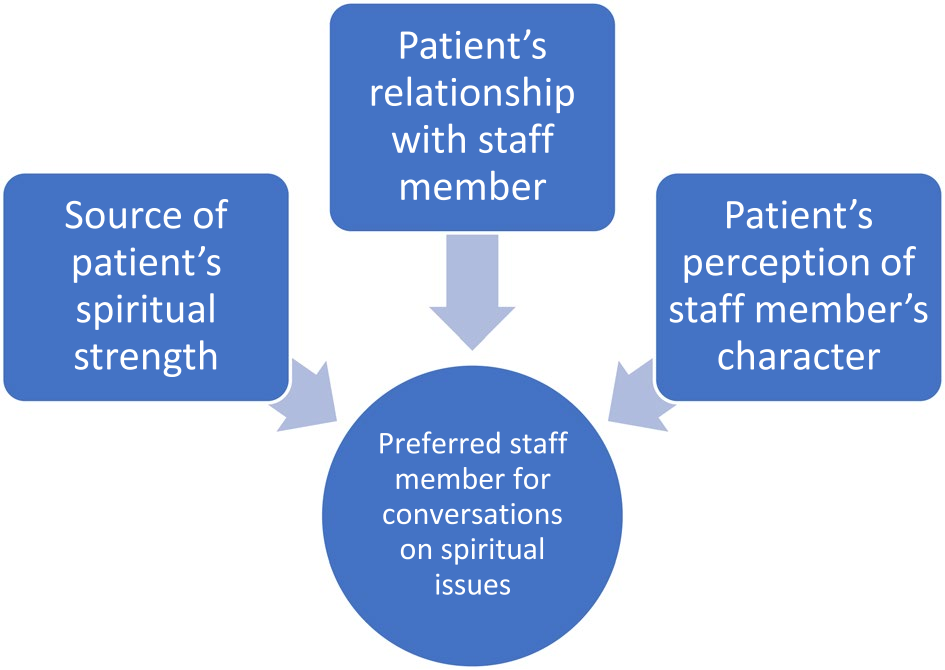
Factors influencing participant’s preference for staff member to discuss spiritual issues

Forty-one participants agreed to be interviewed. Participants expressed a wide range of views. While some participants expressed hesitation to discuss spiritual issues in a healthcare context, this was most often due to the preferred confidante being other than a staff member. For those who were happy to discuss spiritual issues in hospital, preferred staff member was related to the relationship they had with the patient and individual characteristics such as rapport and approachability, familiarity and trust, rather than role of the individual concerned. Themes developed from the transcripts were: (1) Source of spiritual strength; (2) Relationship; and (3) Character. See Fig. 1.

#### Source of Spiritual Strength

Of those who did not want to discuss spiritual issues with staff, most participants reported that they could access spiritual support themselves and therefore hesitated to nominate a staff member with whom they wanted to discuss spiritual issues. Instead, they identified long-standing spiritual companions as the person with whom they preferred to speak, particularly family members and friends. *‘It's not that you're not wanting to talk about it at all. It's just that you have your own—-—support system. Yeah’. (15) ‘I have people who are always messaging me ever since I got here [in hospital]. It’s just giving prayers and stuff like that. From people in my family and friends’. (17)* This was by far the most common reason why respondents did not want to discuss spiritual issues with staff members.* ‘I just don’t think it’s something the staff need to be up on’. (K3)*

In view of this, participants suggested that it would be helpful if, on admission, patients were asked if there was anyone, apart from next of kin, they would like to have contacted if they were very ill for the purpose of providing this support.

Others felt that they would only engage in spiritual discussion with members of their own faith group, or those with whom they shared beliefs: *‘Because they understand my needs’. (K1)* One reason for not engaging with staff was the inability to know if faith was shared: *‘I don’t know them – any of them well enough’. (27)* Some patients expressed reservations about discussing spiritual issues with healthcare staff because they believed spiritual needs were not always present during a hospital admission, and even then some patients would call in an outsider rather than talk to staff: *‘My medical needs are the priority. Maybe religious needs–I ask from Muslim mosque – somebody will come in, big beard’. (25)*

Some patients described their own spiritual experience as focused on drawing on strength within themselves to cope with challenges and therefore being unused to discussing spiritual issues with others: *‘I don’t need to involve anybody else’. (8)* Some patients felt that it was not possible for others to assist with their spiritual needs: *‘I deeply doubt if anyone else can sort them out for you’. (6)*

## Relationship

Most participants felt that discussions about spiritual issues were best conducted within an established therapeutic relationship. This increased the likelihood that the topic would be raised in an appropriate manner at the appropriate time. As a result, patients who were admitted for minor problems which required only a short hospital stay were less likely to think discussions about spirituality were appropriate:* ‘I don’t stay long enough to have a relationship’. (21)*

Those who had been in hospital for some time mentioned the need for a trusting relationship with staff to allow the appropriate approach due to the intimate nature of spirituality: *‘You have to—–—judge the person and you have to tailor the approach to the particular person or personality. I don’t think there’s one – size fits all. You wouldn’t do it the first time you see someone. There’d have to be a little bit of a relationship there. Yeah. Because it’s a – I guess it’s a trust issue as well isn’t it? Because it’s more than medical attention’. (16)*

The existence of a trusting relationship allowed the patient to feel sufficiently safe and supported to discuss difficult issues. *‘They don’t have any other hidden agenda or anything, you know? It’s what they do. It’s their profession to look after you and get you out of here. So to me that’s a safe place. They don’t want anything else – they don’t want anything from you basically’. (16)*

## Character

Apart from those who had had very short admissions, participants overall were very clear on which staff they would talk to about spiritual issues. This was usually not related to the staff role, but the characteristics of the individual. Staff members who made the patient feel comfortable, and whom they could trust, were identified. A minority of participants preferred individuals in a particular staff role to be involved.

### Someone Who Makes Me Feel Comfortable

Patients knew the staff members who made them feel comfortable, though most were not able to articulate why: *‘She just made me feel comfortable. I don't know [how]. It's just the way her personality was. She was just — yeah, she was very — I don't know. She was just nice’. (15) ‘It's just literally, a — uh, maybe a feeling thing. Or, yeah. Hmm, hard to explain. An energy thing’. (31).*

Where this had occurred, participants didn’t mention a particular phrase that was effective in raising spiritual issues. Instead, the topic appeared to occur organically during the conversation: *‘And then they might open up and start talking—and asking stuff. And I just reply. [They may ask]-—- if I'm all right or how — how are you going. Or, is there anything I can get you. You know, you'll tell them’. (23)’… ‘And it was just as simple as – yeah, like I said, “Do you – do you want to talk about how you’re feeling?”’ (14).*

Several attributes of preferred staff were mentioned, such as giving confidence, and relating in a personal way: *‘[The staff are] quite laid back but yet professional—–and they smile but the confidence they – they give -as well so that aura that they give—it’s what makes me feel comfortable’. (9) ‘We were just having a conversation. It was like I am talking to you now. I felt very comfortable with her and it was like talking to a girlfriend. Just a friend. It wasn’t intimidating and she let me babble a lot, so I just talked. I talked about anything like I’m doing now’. (K6) ‘She put me at ease. Just talking about other – normal other things. Something I was interested in… And that’s going to be hard [for staff] because every patient’s different’. (30) ‘We talk the same language’. (27)*

Empathy was also identified as a necessary part of the interaction. When identifying staff with whom they would not want to discuss spiritual issues, patients mentioned staff not being interested or caring about them personally, particularly short-stay patients: *‘These nurses around here are indifferent because they don’t really particularly care about me. They know I’m not going to be staying here very long’. (1)*

Interestingly, other patients excused staff who did not show personal interest, explaining that they were too busy for such conversations: *‘It's just — they've got so much going on, already, with so many people on the floor, I don't know whether — they probably would try to make the time, but I don't think they have the time’. (15)*

Some patients who were inpatients in Roman Catholic hospitals were aware that the increased number of staff members of faith enabled them to provide special comfort: ‘*It’s the camaraderie of Catholicism…because we’re all part of the same club (laughs)’. (19)* Others did not ask to see pastoral care staff as they (mistakenly) thought they were only there to see Roman Catholic patients. In fact, in Australia spiritual care staff are available to all inpatients.

### Someone I Can Trust

Patients described preferred staff members as good listeners who were sincere: *‘I knew they were listening to me, and they took my feelings and thoughts into account with the way they do their job—it would actually – I would be happy to talk to those people’. (11)* ‘*I find personally that if someone asks me a question and they gaze away it’s like okay they’re asking – it’s like someone asking how are you?*—*and not wanting to know how I really feel’. (9)* *‘I know the difference between a pretend smile and a fake one’. (22)*

Trusting that the discussion would be confidential was also an element in deciding which staff member to nominate. For some patients, this meant the spiritual care team, for others it meant psychologists: *‘The psychologist won’t spread anything around. I know that, because she’s told me. But if I say something to a nurse, immediately it will be typed up’. (32)*

### Someone with appropriate expertise

Participants whose first choice of staff was a member of the spiritual care team tended to make this choice because they saw them as the team member with appropriate expertise: *‘They have the education behind them. They have the skill set’. (30) ‘[Since spiritual carers have] a supernatural outlook, with the help of pastoral workers, we can try and see the meaning of suffering, which is part of the human condition, and that can help us to grow humanly, supernaturally, and be, if you like, in control of our illness rather than being overwhelmed by it’. (K4)*

Many respondents commented on the skill of spiritual care workers in listening to the patient and providing encouragement in the hospital setting: *‘He [spiritual carer] was so helpful in just listening to me for so long…It was so great that I was unable to unload all that fear and everything’. (14)*

The non-medical nature of conversations with spiritual carers, as opposed to other staff members, was appreciated in the hospital setting, as well as the availability of ritual such as Holy Communion. Patients were sometimes pleasantly surprised to receive spiritual care services, and appreciated them being offered, even when they weren’t required: *‘I didn’t expect that someone like that would come and talk to me [in hospital]'. (4)*

There was also a suggestion that some patients would like ‘medical’ staff such as doctors and nurses to concentrate more on medical issues*: ‘I prefer them to focus on that’. (16)* However, such patients were happy for these staff members to refer them to spiritual care if they felt it would be of benefit.

Others felt that the role of the doctor gave them the skills to engage in intimate conversations: *‘I think they have – they have a medical obligation to – to disclose things like, so we’ve seen this scan and this could mean that, um, that – that there’s some slightly higher possibility that you’re not a curable patient. I mean they – they need to be able to tell me things like that. And they always manage to be able to tell me things like that very frankly without– I don’t know. They know how to speak, these people’. (18)*

Some patients nominated social workers or psychologists as their preferred staff member. This was also related to their perceived expertise: *‘[Psychologists] deal with that side of the personality. Like, the wellbeing of the mind and soul, whereas a doctor or a nurse, that—to me that’s medical side of things they should look after the medical side. [Psychologists] have the skill set’. (30) [Psychologists] are the kind of people that ask those questions anyway. Like, the more hard hitting deeper questions. And she could ask me more questions like this and I would be more inclined to keep letting her know what’s going on for me’. (31) ‘The social worker is probably the person that you’re closest to, as regards your situation of where you are trying to get to, to get out of this place’. (34)*

However, even when expertise was mentioned, the choice of staff was still associated with the quality of the relationship with the patient: *‘You know, at [another hospital] I didn’t really connect to the psychologist there at all. I didn’t feel the need to chat with them. But this one here, yeah, I did feel more comfortable and feel like I could open up more’. (31)*

## Discussion

In this mixed-methods study, we investigated the preferences of Australian hospital patients regarding which member of staff they would prefer to ask about spiritual issues. Spiritual carers were nominated most frequently. In the qualitative interviews, participants identified spiritual care staff as ‘experts’ in spiritual care. This is consistent with the interprofessional model of spiritual care, based on the biopsychosocial–spiritual care model (Balboni et al., 2014). In this model, all staff members have a role in providing generalist spiritual care, but refer to specialist spiritual care (i.e., chaplains or pastoral care providers) to provide specialist spiritual care when specific needs are identified (Handzo & Koenig, 2004).

One of the hospitals in our study did not have spiritual care staff, and specialist spiritual care needed to be outsourced. Previous studies indicate that generalist staff may have a greater role in spiritual care in this context (Best et al., 2016a, 2016b). This was identified in our cohort, with long-stay rehabilitation patients electing social workers as their preferred staff member. This is a hospital context where they have a more prominent role in patient care and have previously been found to be engaged with the spiritual needs of patients (Jones et al., 2018a, 2018b).

We also found that even in hospitals with specialist spiritual carers, that some spiritual care was given by other staff, which could be due to relationships that exist between patients and the staff they see most often, and with whom they therefore feel most comfortable.

The second preference of participants regarding staff member with whom they would like to discuss spiritual issues was doctors. Many studies have affirmed patients’ views that doctors should play a role in spiritual assessment, at least in crisis situations (Best et al., 2014a, 2014b, 2014c, 2014d, 2015a, 2015b, 2015c, 2015d), which was the context mentioned in our qualitative interviews. Best and colleagues found that advanced cancer patients preferred the doctor to initially engage with spiritual issues in order to build a closer relationship and to provide personalised holistic care in line with the patient’s values. Patients did not want the doctor to give spiritual advice, and if spiritual needs arose, expected to be referred to spiritual care staff, once again in line with the interprofessional model of spiritual care (Best et al., 2014a). Research in Australia has demonstrated advantages for patient care when doctors and spiritual carers collaborate (Carey & Cohen, 2009).

In these extreme situations of serious illness and self-awareness the locus of spirituality and medical concerns are brought closer together (Mesquita et al., 2017). In the crisis situation, spiritual conversation provides leverage or opens a door to ask difficult medical questions that otherwise might be avoided, such as those about life expectancy, effect of treatment on lucidity, and when to call the family (Best et al., 2014a, 2014b, 2014c, 2014d). It could be that in less serious situations, spirituality and medical care can be more easily compartmentalised and therefore directed to other HCP (Jones et al., 2018a, 2018b).

Certainly, in less acute medical settings, patients may not consider a doctor’s role in spiritual assessment or care to be vital. In one study, only 10% of a sample of internal medicine clinic patients would discuss spiritual issues in lieu of discussing medical problems with their doctors (MacLean et al., 2003). This was reflected in the attitude expressed by this cohort that spiritual issues are less likely to be present if illness is not severe (Best et al., 2022a, 2022b).

It is interesting that nurses were not nominated as a preference by this cohort, as the primary caregiver in the inpatient context. In our study, nurses were prominent in participant narratives of spiritual care, and it is possible that there was a misunderstanding in what constitutes spiritual care. Spiritual care has long been recognised as a role for the nurse (International Council of Nurses, 2012).

Previous studies have demonstrated poor concordance between patients and HCP regarding frequency of spiritual discussions (Ellis, 2013; Epstein-Peterson et al., 2015; Ford et al., 2014). It is possible, in a secular community like Australia where spiritual matters are not often discussed, that the addressing of spiritual needs is understood as just part of holistic care without realisation of its existential nature. One of our participants did not distinguish between psychological and existential needs. As such, language may be the issue and future research should explore this area.

While a small minority of patients in this cohort did not want to be asked about spiritual issues, the qualitative data suggest that this was most frequently due to those individuals having alternative avenues for discussion which were previously established. This finding was reported in a literature review which found that patients who looked to others for spiritual support did not want to be asked about their spirituality by healthcare staff (Best et al., 2015a, 2015b, 2015c, 2015d).

Despite our attempts to identify the clinician which patients prefer for spiritual discussions, it was clear from our qualitative data that it is individual characteristics to which patients respond, rather than clinical role. Staff who make patients feel comfortable, who are empathic, good listeners and authentic in their care of the patient were described as ones who were most effective in this role.

This was reported in a Danish study, where hospice patients reported that a bodily and non-verbal dimension of their interaction with staff, such as sitting next to the patient and using touch, affected their interpretation of the HCP’s availability and their perception of connectedness (Voetmann et al., 2022) In this study, patients were also found to communicate unspoken signals to HCPs for their interpretation and response. In responding, HCPs allowed patients to feel seen and understood and willing to engage in conversations of a spiritual nature.

The nature of this quality of the HCP, while obviously important, was not clear. A review by Paal and colleagues (2015) found that developing the sensitivity of staff to their own spirituality was the most crucial step in developing spiritual care skills; and that providing spiritual care was not only about posing the ‘correct’ questions, but also about listening, being present, available, and free from stereotypes towards different cultures and religions (Paal et al., 2015). This is also consistent with findings that discussion about spirituality requires advanced communication skills (Ford et al., 2014).

Some participants in our study assumed (incorrectly) that the spiritual care team in Catholic hospitals only cared for Catholic patients, while some Catholic patients expressed an affinity for Catholic staff. The role of faith concordance between the patient and spiritual care provider is important to address in a pluralistic society like Australia. Some authors have described religious and cultural diversity as a challenge, with potential risks including the HCP inadvertently imposing their beliefs on clients or offending them when conflicts in belief systems emerge (Best et al., 2015a; Hodge & Lietz, 2014). As such, efforts have been made to provide the special skills required (Ganzevoort et al., 2014).

However, a Dutch study found that patients in faith-concordant encounters and faith-discordant encounters evaluated spiritual care encounters with equal positivity (Liefbroer & Nagel, 2021). This may reflect the growing professionalisation of chaplaincy enabling practitioners to engage with all patients regardless of faith (Pesut, 2016; Stifoss-Hanssen et al., 2019). A review of studies examining spiritual discussions between doctors and patients found that, although faith discordance was a barrier, it could be overcome with a mutual ecumenical perspective and/or physicians’ communication skills (Best et al., 2016a, 2016b). This suggests that spiritual care training for HCPs should address this issue, particularly in a pluralist setting.

The content of HCP discomfort has not been fully explored. Perhaps some HCPs do not have the personal resources required for such intimate conversations, which are known to require connecting with others (Daaleman et al., 2008; Rumbold, 2002) and being uncomfortable with uncertainty (Best et al., 2015a; Daaleman et al., 2008; Jones, 1999; Puchalski et al., 2006). Our study supports this finding, with those staff able to identify the right timing for spiritual enquiry and listen carefully to responses in a patient-centred way recognised by patients as the most successful spiritual carers by our participants.

This finding also suggests the need to ensure that all healthcare disciplines receive training in spiritual care, of which an important step is the development of the reflective capacity of staff to consider the importance of the spiritual dimension in their own life, and communication skills, including awareness of the non-verbal aspects of communication in the HCP–patient relationship.

Staff training is also needed for the interprofessional model of spiritual care to operate effectively. All staff need to be empowered to include spiritual care in patient care, cognizant of their own spirituality, and made aware of indicators for pastoral care referral when they feel specialist spiritual care is required (Jones et al., 2021a, 2021b). A study of palliative care consultations found that spiritual issues were discussed more often if the doctor raised the topic (Best et al., 2019), which further suggests that if staff were trained in spiritual care conversations, patients’ needs are more likely to be addressed.

Lack of training in spiritual care has been identified as an important barrier to the provision of spiritual care in healthcare (Best et al., 2015b; Jones et al., 2018a, 2018b). Ways to address this are increasingly available (Best et al., 2020; Puchalski et al., 2020) and recognised by stakeholders and policy makers as important (Holmes, 2018; Spiritual Health Association, 2020). Institutional support of such programmes is needed to maximise success (Anandarajah et al., 2016; Brémault-Phillips et al., 2015; Daudt et al., 2019; Jones et al., 2021a, 2021b), an example of which was seen in the Roman Catholic institutions in this study. Avenues for future study include determining whether the religious or secular nature of the establishment may or may not influence the practice of spiritual care.

### Limitations

Non-English speaking and seriously ill patients were excluded from this study, and their needs may differ from those included. We did not collect data to compare participants with non-participants, and we cannot therefore be sure that all views were represented; however, as our response rate was 95%, we are confident that our results report the majority view. Qualitative analysis is not intended to be generalisable and other cohorts may respond differently. Furthermore, most of the hospitals included in the study were faith-based institutions, which may have impacted the practice of spiritual care.

This was a unique exploratory study examining patients’ preferred staff members for discussing spiritual issues in the hospital ward. While we looked for associations between participant spirituality/religiosity, spiritual wellbeing and staff preferences, effect sizes were small. Future research should further investigate patient views with other validated religious/spiritual measurement instruments.

## Conclusions

In conclusion, this study of a heterogeneous group of Australian hospital patients found that the first staff preference for discussing spiritual issues in healthcare was spiritual care staff, followed by doctors. However, the clinician’s personal characteristics were more important than professional role when patients decide who they will speak to on this intimate topic. Our findings confirm that all HCP can have a role in spiritual care of patients, but that training is required to ensure that patient needs are appropriately met.
